# Relationship between anterior segment biometry and primary angle-closure glaucoma in Hubei region: a retrospective case-control study

**DOI:** 10.3389/fmed.2024.1418566

**Published:** 2024-08-23

**Authors:** Shan Wang, Han Zhang, Wanju Yang, Ying Zhang, Hui Qin, Man Wang, Xinlan Lei, Kuiliang Yang, Hong Zhang, Yiqiao Xing

**Affiliations:** ^1^Aier Eye Hospital of Wuhan University, Wuhan, Hubei, China; ^2^Eye Center, Renmin Hospital of Wuhan University, Wuhan, Hubei, China

**Keywords:** primary angle-closure glaucoma, IOL-master 700, anterior chamber depth, white-to-white, lens thickness, central corneal thickness, axial length

## Abstract

**Objective:**

To investigate the pathogenesis of Primary Angle-Closure Glaucoma (PACG) and its relationship with the anatomical structure of the anterior segment by obtaining biometric parameters using the IOL-Master 700.

**Methods:**

A retrospective case–control study was conducted. Clinical data from 39 PACG patients and 40 normal controls treated at the Aier Eye Hospital affiliated with Wuhan University from January to December 2022 were collected. Anterior chamber depth (AC), white-to-white (WTW), lens thickness (LT), central corneal thickness (CCT), axial length (AL), corneal curvature (K1), corneal curvature (K2), and lens position (LP) were measured using the IOL-Master 700 to analyze the characteristics and differences in the anterior segment structure of both groups. Statistical methods included independent sample t-tests and logistic regression analysis.

**Results:**

Significant differences were found in the anterior segment biometric parameters between PACG patients and normal controls (*p* < 0.05). Anterior chamber depth, white-to-white, lens thickness, central corneal thickness, axial length, and K2 were all related to the occurrence of PACG (*p* < 0.05). The occurrence of PACG was negatively correlated with ACD, CCT, and AL (OR = 0.12–0.64, *p* < 0.05), and positively correlated with LT.

**Conclusion:**

Compared to the normal control group, PACG patients in the Hubei region have a smaller anterior segment space, narrower angles, thicker lens, thinner cornea, shorter axial length, flatter cornea, and more anteriorly positioned lens.

## 1 Introduction

Primary angle-closure glaucoma (PACG) is a prevalent form of glaucoma that has a higher risk of causing bilateral blindness compared to primary open-angle glaucoma or secondary glaucoma (1). The incidence of PACG varies significantly among different regions and ethnicities, with Asia being a high-incidence area and a major cause of vision loss in East Asian populations (2). Patients with PACG often present with multiple anatomical risk factors, such as a smaller cornea, shallow anterior chamber, shorter axial length (AL), thicker lens, pupillary block, and iris configuration (1, 3, 4). Recent advancements in ophthalmic imaging technology have enhanced our understanding of the relationship between anterior segment biometric parameters and PACG. It has been found that, apart from the common parameters mentioned above, factors like lens position and relative lens position also contribute to the development and progression of PACG (5, 6).

The IOL-Master 700 (Carl Zeiss, Germany), is an innovative non-invasive optical biometer that utilizes swept source optical coherence tomography (SS-OCT) with a 1,050 nm wavelength. This device efficiently captures three-dimensional anterior segment data of eye tissue with high lateral and axial resolution. By providing longitudinal sections of optical interfaces and determining the true optic axis length through imaging the fovea centralis, the IOL-Master 700 offers detailed information on corneal anterior and posterior surfaces, lens anterior and posterior surfaces, macular neuroepithelium, and pigment epithelium interface (7, 8). It accurately measures parameters such as anterior chamber depth (ACD), lens thickness, and vitreous length. These anterior segment biometrics obtained by the IOL-Master 700 are superior to those obtained by other non-contact biometric devices (9, 10).

Previous research on the anatomical structure of the anterior segment in individuals with Primary Angle-Closure Glaucoma (PACG) has primarily utilized manual measurements from anterior segment Optical Coherence Tomography (OCT) and Ultrasound Biomicroscopy (UBM), which suffer from poor reliability and repeatability. This study utilizes the IOL Master 700 to conduct precise biometric measurements of ocular parameters in PACG patients, examining the characteristics of anterior segment biometric parameters in the Hubei region and investigating the relationship between the onset of PACG and the anatomical structure of the anterior segment.

## 2 Methods

This retrospective case–control study focused on patients diagnosed with Primary Angle-Closure Glaucoma (PACG) and normal controls who were treated at the Aier Eye Hospital affiliated with Wuhan University between January 2022 and December 2022 (11). The inclusion and exclusion criteria were carefully defined.

### 2.1 Inclusion criteria

For PACG patients, as outlined in the Chinese Glaucoma Guidelines (2020), include static gonioscopy findings of iris trabecular contact (ITC) over an area greater than 180°, accompanied by elevated intraocular pressure or peripheral anterior synechiae (PAS), with or without glaucomatous optic nerve changes. SACG-ASL refers to abnormal ciliary zonules causing narrowing or closure of the anterior chamber angle, leading to elevated intraocular pressure, with or without glaucomatous disc changes and visual field impairment. Normal controls are defined as patients with no history of ocular diseases or surgeries, normal intraocular pressure (IOP < 21 mmHg), open angles (≥270°) upon static or dynamic gonioscopy, normal optic nerve (cup-to-disk ratio < 0.5 or asymmetry <0.1, without disc hemorrhage, pallor, or other abnormalities), and normal visual field (no visual field defects).

### 2.2 Exclusion criteria

Patients with primary angle-closure glaucoma (PACG) must meet the following conditions: (a) History of other ocular diseases or surgeries, such as cataract, glaucoma, retinal diseases, ocular trauma, etc. (b) Systemic diseases or medication history, such as diabetes, hypertension, heart disease, kidney disease, thyroid disorders, steroids, etc. (c) Family history, such as first-degree relatives with PACG.

Normal controls: patients without PACG must meet the following conditions: (a) History of ocular diseases or surgeries, such as cataract, glaucoma, retinal diseases, ocular trauma, etc. (b) Systemic diseases or medication history, such as diabetes, hypertension, heart disease, kidney disease, thyroid disorders, steroids, etc. (c) Family history, such as first-degree relatives with PACG.

### 2.3 Research methods

Ocular examination: the anterior chamber angle of the patient is assessed independently by two experienced ophthalmologists utilizing a static gonioscope. Consensus will be reached only when both practitioners concur. Given the potential presence of corneal edema in patients with primary angle-closure glaucoma (PACG), intraocular pressure is evaluated in all patients utilizing the Icare tonometer (Icare ic100, Finland), with five measurements taken for each eye and the average value recorded.

The study involved biometric parameter measurements of the anterior segment using the IOLMaster 700, a Carl Zeiss ophthalmic imaging device from Germany. The device was operated by a skilled technician following a standardized procedure to ensure precision and consistency. Participants were seated and instructed to keep their eyes naturally open, fixate on a specific point on the device, and maintain their head and eye position without blinking during the measurement process. Each eye underwent three measurements, and the average value was recorded as the final result. The measured biometric parameters of the anterior segment included: anterior chamber depth (AC), white-to-white (WTW), lens thickness (LT), central corneal thickness (CCT), axial length (AL), corneal curvature (K1), corneal curvature (K2), and lens position (LP), and relative lens position (RLP). ACD refers to the distance from the corneal endothelium to the anterior lens surface, WTW is the horizontal distance between the iris roots, LT is the distance from the anterior to the posterior lens surface, CCT is the distance from the anterior to the posterior corneal surface, AL is the distance from the anterior corneal surface to the retinal pigment epithelium, K1 and K2 represent the maximum and minimum corneal curvature values, LP is the distance from the posterior corneal surface to the anterior lens surface, and RLP is the ratio of LP to AL.

### 2.4 Statistical methods

Statistical analysis was performed using SPSS(IBM) software version 22.0. An independent samples *t*-test was utilized to compare continuous variables between the FACG group and the normal control group. Following this, univariate and multivariate logistic regression analyses were carried out to identify significant influencing parameters for the occurrence of PACG in the Hubei region (12). A *p* value less than 0.05 was considered statistically significant.

## 3 Results

### 3.1 General information and clinical data

This study involved 39 patients (76 eyes) diagnosed with Primary Angle-Closure Glaucoma (PACG) and 40 normal controls (64 eyes). There were no statistically significant differences in age and gender between the PACG patients and normal control subjects (*p* > 0.05). The mean intraocular pressure (IOP) among patients with primary angle-closure glaucoma (PACG) is 28.07 ± 4.87, whereas the mean IOP for the control group is 13.08 ± 2.57.

### 3.2 Comparison of anterior segment measurement parameters

There were statistically significant differences between the PACG group and the normal control group in terms of anterior chamber depth, white-to-white (WTW), lens thickness (LT), central corneal thickness (CCT), axial length (AL), corneal curvature K1, corneal curvature K2, and lens position (*p* < 0.05). Compared to the normal control group, PACG patients had a shallower anterior chamber, shorter WTW, thicker lens, shorter AL, smaller corneal curvature K1 and K2, and a more anterior lens position (Table 1).

**Table 1:** Comparison of anterior segment biometric parameters between PACG patients and normal controls.

Value	Normal (*n* = 40)	PACG (*n* = 39)	*t*	*p*
ACD (mm)	3.11 ± 0.26	2.33 ± 0.30	14.95	<0.05
WTW (mm)	11.88 ± 0.33	11.47 ± 0.44	5.64	<0.05
LT (mm)	4.34 ± 0.28	4.77 ± 0.61	4.87	<0.05
CCT (mm)	535.30 ± 26.34	536.52 ± 37.43	0.22	0.04
AL (mm)	23.16 ± 0.78	22.32 ± 1.14	4.63	<0.05
K1 (D)	43.41 ± 1.63	44.14 ± 1.63	2.51	0.01
K2 (D)	44.09 ± 1.58	45.12 ± 1.61	3.59	<0.05
LP	5.27 ± 0.25	4.72 ± 0.34	10.17	<0.05

ACD, Anterior chamber depth; WTW, White-to-White; LT, Lens thickness; CCT, Central corneal thickness; AL, Axial length; K1, K2, Keratometry; LP, Lens position; RLP, Relative lens position.

### 3.3 Analysis of related factors univariate logistic regression analysis

Adjusted for age and gender, it was found that anterior chamber depth (*p* < 0.05), white to white distance (*p* < 0.05), lens thickness (*p* < 0.05), central corneal thickness (*p* < 0.05), axial length (*p* < 0.05), and K2 (*p* < 0.05) were all associated with the occurrence of primary angle-closure glaucoma (PACG) (after Bonferroni correction) as shown in Table 2. The presence of PACG showed a negative correlation with anterior chamber depth, central corneal thickness, and axial length (OR = 0.12–0.64, *p* < 0.05), and a positive correlation with lens thickness. However, in the multivariate logistic regression analysis, none of the anterior segment parameters showed statistical significance.

**Table 2:** Univariate logistic regression analysis of anterior segment parameters related to primary angle-closure glaucoma.

Value	OR	95%CI	*p*
ACD (mm)	0.12	0.02 ~ 0.60	<0.05
WTW (mm)	0.02	0.43 ~ 2.53	<0.05
LT (mm)	1.90	1.37 ~ 2.33	<0.05
CCT (mm)	0.64	0.28 ~ 0.99	<0.05
AL (mm)	0.13	0.62 ~ 0.27	<0.05
K1 (D)	1.23	0.97 ~ 1.48	0.02
K2 (D)	1.36	1.02 ~ 1.72	<0.05
LP	0.23	0.07 ~ 2.43	0.75

ACD, Anterior chamber depth; WTW, White-to-White; LT, Lens thickness; CCT, Central corneal thickness; AL, Axial length; K1, K2, Keratometry; LP, Lens position; RLP, Relative lens position.

## 4 Discussion

This study utilized the IOL Master 700 to compare the biometric parameter differences between normal subjects and patients with Primary Angle-Closure Glaucoma (PACG). Significant variations were noted in the anterior segment biometric parameters, with PACG patients exhibiting shallower anterior chamber depth, shorter white-to-white (WTW) distance, thinner central corneal thickness (CCT), and shorter axial length (AL) compared to the control group. Additionally, PACG patients had a greater lens thickness (LT).

Patients with Primary Angle-Closure Glaucoma (PACG) exhibit a crowded anterior segment structure, shorter axial length, shallower anterior chamber depth, and thicker lens. Our study’s analysis demonstrated that individuals with PACG have significantly thicker lenses compared to normal controls, with each 1 mm increase in lens thickness correlating to a 1.90 times higher risk of developing PACG. These structural characteristics predispose PACG patients to narrower angles and more frequent blockage of the trabecular meshwork by the peripheral iris. The increased lens thickness causes the anterior lens capsule to exert pressure on the iris sphincter muscle, resulting in pupil blockage. This obstruction disrupts the flow of aqueous humor from the posterior to the anterior chamber, leading to its accumulation in the posterior chamber. The accumulated aqueous humor further contributes to the forward bulging of the iris, narrowing the angle and potentially causing complete closure, ultimately triggering the onset of PACG. Logistic regression analysis confirmed that an increase in lens thickness significantly raises the risk of PACG. A longitudinal study tracking a female patient with bilateral PACG over 12 years, monitoring changes in lens thickness, revealed an annual increase in lens thickness well above the average for normal individuals (13). The thickening of the lens in PACG patients results in a shallower anterior chamber and crowding, which, in conjunction with ongoing thickening, causes the iris to bulge forward, leading to pupil blockage, hindering aqueous humor drainage, and causing a rapid rise in intraocular pressure. Moreover, due to the persistent thickening of the lens, many patients who solely undergo trabeculectomy experience progressive angle narrowing, leading to recurrent PACG attacks (14). Therefore, multiple studies suggest that early cataract surgery for glaucoma patients can slow down or postpone the onset of the disease (15–17). The results of this study show that if the thickened lens is not removed in time, the risk of a PACG attack is high. Hence, prophylactic cataract surgery should be considered for PACG patients.

Previous research has indicated that primary angle-closure glaucoma (PACG) patients typically exhibit smaller corneal diameters and curvatures compared to individuals without the condition. A study comparing acute, subacute, and chronic PACG patients to normal individuals revealed that all PACG groups displayed smaller corneal diameters and curvature radii than the normal group, with the most significant differences observed between the acute PACG group and the normal group. Additionally, the study found that the K2 values of the PACG patient group were higher than those of the normal control group, indicating a greater curvature of the steepest meridian. This difference may be attributed to the older age distribution within the study population. Notably, against-the-rule astigmatism, characterized by higher K2 values than K1 values, is common among the elderly, suggesting a higher proportion of older individuals within the PACG patient population.

The novelty of this study lies in the use of the IOLMaster 700 for measuring anterior segment biometric parameters. The IOLMaster is a non-contact, non-invasive, and visual biometry instrument. Current research predominantly employs manual measurements through anterior segment Optical Coherence Tomography (OCT) and Ultrasound Biomicroscopy (UBM), which lack reliability and repeatability. The IOLMaster 700 incorporates the latest global Swept-Source OCT technology into biometry, enabling not only rapid and precise measurement of various ocular biometric parameters but also the presentation of full axial OCT tomographic images, allowing for the visual assessment of measurement accuracy. This ensures the acquisition of accurate biometric parameters of the anterior segment in patients with Primary Angle-Closure Glaucoma (PACG), resulting in high patient compliance.

The study’s limitations stem from its retrospective case–control design, which could introduce selection bias and confounding factors. It did not thoroughly explore potential influences on Primary Angle-Closure Glaucoma (PACG) incidence, such as genetic, environmental, and lifestyle factors. Additionally, the purpose of this study was to investigate the relationship between anterior segment anatomical structures and PACG. Therefore, we adopted an inclusive definition that encompassed all patients with a risk of angle closure. This was done to ensure that we could capture all patient groups that might benefit from further diagnosis and treatment, regardless of whether they presented with primary angle closure (PAC) or PACG. Moreover, the small sample size limited to Hubei patients may not be representative of the broader PACG population. The lack of multicenter or large-sample studies could impact result stability and generalizability. Future research should prioritize prospective cohort studies or randomized controlled trials, expand sample sizes and geographic diversity, control for confounders, validate current findings, and deepen understanding of PACG pathogenesis for more robust evidence on prevention and treatment.

In conclusion, this study underscores the significant disparities in anterior segment biometric parameters between PACG patients and normal controls, reinforcing the notion that PACG pathogenesis is intricately linked to anomalies in the anterior segment’s anatomical configuration. Our findings reveal that parameters such as anterior chamber depth (ACD), white-to-white (WTW) distance, central corneal thickness (CCT), axial length (AL), keratometry readings K1 and K2, and lens position (LP) are considerably reduced in PACG patients, while lens thickness (LT) is markedly increased. These results align with prior research, delineating a characteristic profile for PACG patients: a constricted anterior segment space, narrowed angles, an augmented lens, a diminished cornea, a truncated axial length, a flatter cornea, and a lens positioned more anteriorly. The aberrations in these parameters elucidate the impediments to aqueous humor outflow in PACG, primarily due to the forward displacement and thickening of the lens, which precipitates a decrease in anterior chamber depth and width, angle closure, elevated intraocular pressure, optic nerve damage, and visual field impairments. Therefore, the assessment of anterior segment biometric parameters in PACG patients plays a crucial role in evaluating the risk of developing PACG, enabling early identification and intervention.

## Data Availability

The raw data supporting the conclusions of this article will be made available by the authors, without undue reservation.
